# Temporal properties of direct current sensory block of the rat sciatic nerve using the C-fiber reflex

**DOI:** 10.1088/1741-2552/ae2e89

**Published:** 2025-12-30

**Authors:** David B Green, Shane A Bender, Varun S Thakkar, Thomas E Love, Hannah E Hill, Kevin L Kilgore, Niloy Bhadra, Tina L Vrabec

**Affiliations:** 1 Department of Physical Medicine and Rehabilitation, The MetroHealth System, Cleveland, OH 44109, United States of America; 2 Department of Physical Medicine and Rehabilitation, School of Medicine, Case Western Reserve University, Cleveland, OH 44106, United States of America; 3 Department of Medicine, School of Medicine, Case Western Reserve University, Cleveland, OH 44109, United States of America; 4 Population Health and Equity Research Institute, The MetroHealth System, Cleveland, OH 44109, United States of America; 5 Department of Population and Quantitative Health Sciences, Case Western Reserve University, Cleveland, OH 44106, United States of America; 6 Department of Orthopedics, The MetroHealth System, Cleveland, OH 44109, United States of America; 7 Louis Stokes VA Medical Center, 10701 East Boulevard, Cleveland, OH 44106, United States of America

**Keywords:** electrical nerve block, sensory nerve block, direct current, pain

## Abstract

**Objective.:**

Direct current (DC) electrical block of peripheral sensory axons has potential for clinical applications in pain management. The C-fiber reflex (CFR), elicited via noxious stimulation of the foot, is suitable for investigating the activation of unmyelinated C-fiber nerves, the fiber class that is responsible for lingering pain sensations.

**Approach.:**

In anesthetized rats, the CFR was elicited via electrical stimulation to the plantar surface of the hindpaw, and the resulting C-fiber-evoked electromyography (EMG) signals were recorded from the ipsilateral biceps femoris muscle. A carbon separated interface nerve electrode was used to deliver DC block to arrest action potentials in the sciatic nerve. The efficacy of the block was observed as a reduction/abolition of the magnitude of the EMG in a time window corresponding to the latency of C-fibers activity.

**Main results.:**

Complete cessation of nerve activity could be achieved instantaneously by applying DC at the ‘block threshold (BT)’. At amplitudes below the BT, complete block could be induced over a period of seconds to minutes, with lower currents being correlated with longer induction times. When block was applied for prolonged periods of time, block was sustained following the cessation of DC delivery. This ‘recovery period’ was longer for longer durations of block application.

**Significance.:**

The CFR is an established method to investigate pharmaceutical pain therapies, yet to date, has not been used to assess electrical block of sensory axons. Therefore, anatomical and electrophysiological methods were used to validate this method. DC nerve block shows promise for clinical pain management applications. Furthermore, the temporal properties described here could be used to reduce overall electrical current delivery and improve safety.

## 1. Introduction

Direct current (DC) electrical nerve block has been demonstrated to effectively block motor [1–4], autonomic [5],and sensory [6, 7] nerve conduction *in-vivo*. Until recently, DC nerve block has been restricted to investigational scientific research due to its potential to cause permanent damage to nerves and surrounding tissues [8]. However, several technological advances have been developed that allow DC to be applied safely for several hours [1, 9]. These allow the possibility of using DC nerve block in chronic clinical applications to treat conditions such as spasticity, cardiac arrhythmias, and both acute and chronic pain of peripheral origin. Furthermore, DC nerve block has advantages over existing pharmacological nerve block approaches: it can be turned on and off rapidly, it is gradable, and has no systemic side effects [1, 5].

Previous research has characterized two temporal phenomena exhibited in DC nerve block:

### The induction effect:

A specific amplitude of DC will instantaneously block all axonal conduction. This value is termed the instantaneous block threshold (BT_0_). A lower amplitude can instantly block only a subset of axons. However, over time (ranging from seconds to minutes) the block effect can steadily increase until all axons are blocked [4, 5, 7]. This was termed the Induction Effect [1].

### The recovery effect:

A sustained, carry-over nerve block can persist from seconds to minutes after the DC current is terminated. The time of recovery depends on several factors, such as the percentage of achieved block, and the duration of the applied current [1, 4, 5].

In the present study, the rat sciatic nerve was used to investigate DC block of sensory nerve conduction. The sciatic nerve is a mixed motor and sensory nerve that extends from mostly from lumbar levels 4 and 5 (and to a lesser extent from levels 3 and 6) of the spinal cord [10]. At mid-thigh level it branches into the common peroneal, anterior tibial, and sural nerves. These three nerves provide the majority of the sensory innervation to the foot [11]. Note that the saphenous nerve also innervates the foot but does not originate from the sciatic nerve. The saphenous was cut at the beginning of all experiments so that the effect of DC block of the sciatic could be examined in isolation.

A number of techniques have been employed to investigate the extent of activation of the peripheral sensory nervous system in rodents [6, 7]. Slowly conducting, unmyelinated C-fibers are responsible for lingering pain sensations, as well as the chronic pain suffered in neuropathic disorders [12]. For this reason, the C-fiber reflex (CFR) electromyography (EMG) technique was chosen. This electrophysiological technique allows the assessment of C-fiber activity in the whole of the sciatic nerve, while remaining minimally invasive. However, artifact contamination must be carefully assessed.

The CFR (as it is known in animal studies [13–15]), also known at the flexion or withdrawal reflex (from clinical literature [16, 17]), is a physiological reflex that allows for a limb to be withdrawn from a noxious stimulus. The CFR can be elicited from all four limbs in human [18]. It is a polysynaptic response that is processed by the spinal cord as it is still present in spinalized rats [13, 19]. Experimentally, the reflex can be elicited in rats and humans by electrical stimulation of afferent fibers in either the plantar surface of the foot or the sural nerve [13, 20, 21]. Previous studies have characterized the latency of the C-fiber response in acute rodent studies [13, 15, 22–24]. It has also been established that C-fibers windup can be mitigated by maintaining the stimulation rate lower than 0.3 Hz [15, 25–31]. We tested this property in our setup and confirmed that the use of appropriate stimulation frequencies prevents the introduction of C-fiber windup in our experimental protocol.

**Figure 1.**
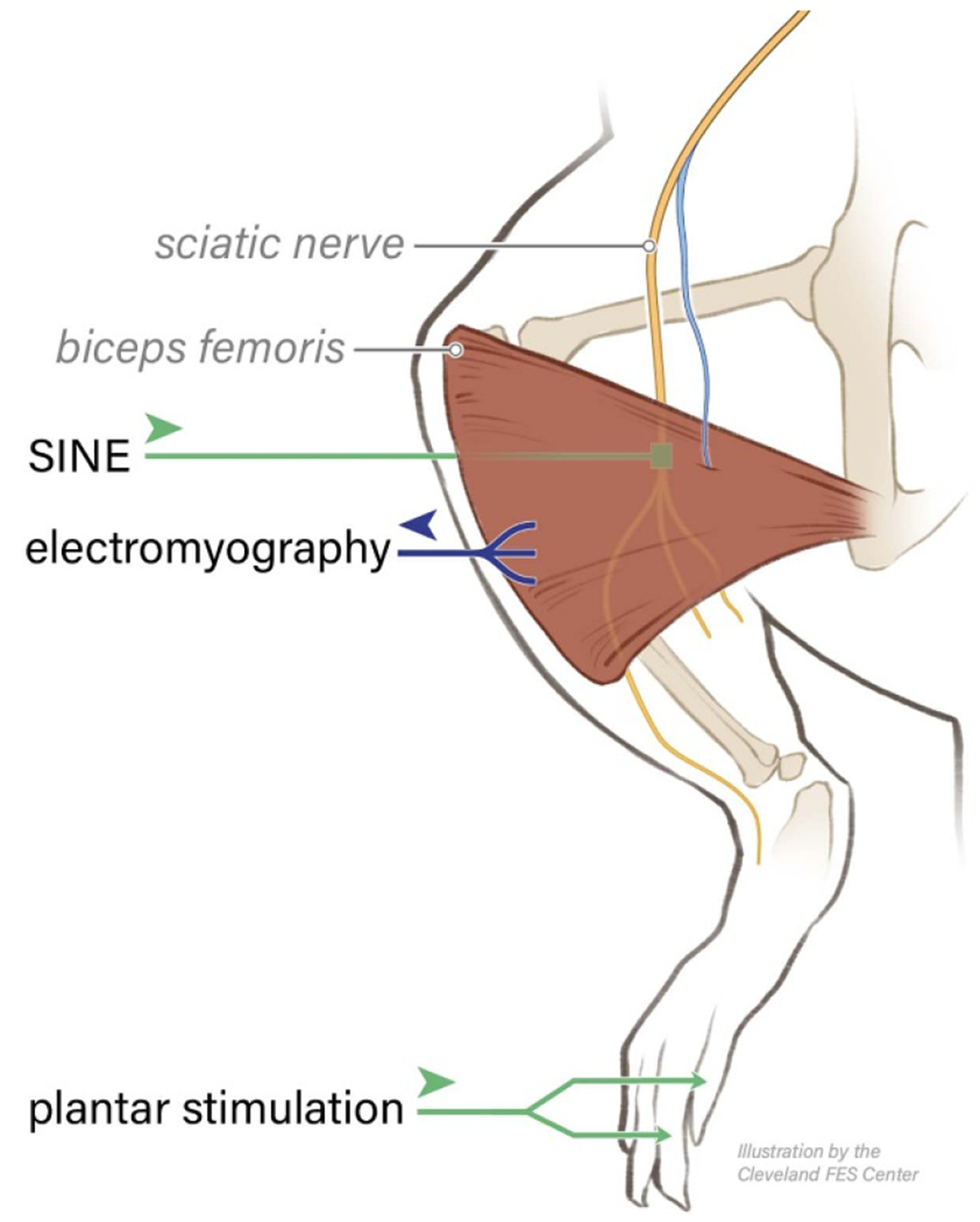
*In-vivo* experimental setup (rat hind limb in prone position): Plantar stimulation activates nociceptive C-fibers, whose action potentials travel along the sciatic nerve, through the SINE blocking cuff, toward the spinal cord. The reflex motor impulses are generated in the spinal cord, and the resulting EMG signals are recorded in the biceps femoris. The sciatic nerve branch that innervates the biceps femoris is shown in blue.

The CFR can be quantified by recording electromyography (EMG) signals from the biceps femoris in anesthetized rats or awake humans (figure 1). Different levels of stimulation elicit different reflex responses. Low, non-noxious stimulation produces a short duration, short latency EMG component, caused by activation of myelinated sensory fibers that are involved in transmission of non-nociceptive tactile sensation. Higher stimulation amplitudes are noxious, activating unmyelinated C-fibers and produce and additional higher latency, longer duration EMG component [13, 20]. In humans, the amplitude of the C-fiber-component of the EMG signal correlates strongly with perceived pain [16, 18]. In tandem, both the C-fiber-related EMG and perceived pain level can be increased by injection of noxious agents such as capsaicin, or decreased by analgesics such as morphine or acetaminophen in a dose-dependent manner [18].

Prior to the assessment of the temporal properties, the model was evaluated to verify that the experimental setup would be free of spurious electrical effects. This *in-vivo* model was then used to demonstrate the effectiveness of DC nerve block on nociceptive fibers and characterize the temporal properties of induction and recovery.

## 2. Methods

### Animals:

All animal experiments were approved by the Institutional Animal Care and Use Committee of Case Western Reserve University. CWRU is an AAALAC accredited institution and conforms to relevant federal, state, and local laws and regulations, as well as institutional policies. Our team was trained and certified by CWRU staff and adheres to Public Health Service policy and the Guide for the Care and Use of Laboratory Animals (8th edition) [32] when working on IACUC-approved protocols. Male adult Sprague–Dawley rats (Charles River, Raleigh, NC) weighing 400–500 grams, were maintained under ketamine/xylazine anesthesia (8.6 mg ml^−1^ ketamine and 0.28 mg ml^−1^ xylazine) infused intravenously (1000–1500 *μ*l h^−1^), with the addition of 0.5% inhaled isoflurane. This combination resulted in a consistent and robust EMG response. The depth of anesthesia was assessed by monitoring the hindpaw pinch reflex, the eye blink reflex, and heart rate. The anesthesia was adjusted such that both reflexes were abolished whilst allowing a robust EMG signal to be recorded with a minimum signal-to-noise ratio of 3.

### Surgery:

A small skin incision was made in the inner left thigh to access the saphenous nerve. The nerve was separated from the femoral artery and vein, then cut. Another small skin incision was made at the caudal edge of the left biceps femoris, and blunt dissection was used to clear the connective tissue securing this edge of the muscle. Reaching under the biceps femoris, further blunt dissection was used to free the sciatic nerve, allowing placement of the carbon separated interface nerve electrode (CSINE) (described below).

### Foot stimulation to elicit CFR:

Two stainless steel needle electrodes (13 mm length, 0.4 mm diameter, Rhythmlink, Columbia, SC, USA) were inserted subcutaneously into the plantar surface of the left foot: on the lateral side of the 5th and medial side of the 4th digits to preferentially stimulate the sural nerve receptive field [11, 13]. A noxious electrical stimulation was applied using a Grass S88 stimulator and PSIU8 stimulus isolation unit (Grass Technologies, RI, USA) to deliver monophasic square pulses at 5–15 mA, 10–50 ms, rate 0.1 Hz.

### Electromyography recording:

Three 5 mm stainless steel needle electrodes (Rhythmlink, Columbia, SC, USA) were inserted in the left biceps femoris. The signal was amplified using a CED 1902-10 isolated head stage (10× gain) and amplifier (CED 1902, 1000× gain), then digitized using a 1401 ADC into Spike2 software (version 10) with a sample rate of 20 kHz (Cambridge Electronic Design, Cambridge, UK). The signals were AC coupled to the amplifier, and low-pass filtered at 10 kHz. A 60 Hz notch filter, as well as an additional powerline noise reduction device was used to reduce mains interference (Hum Bug, Digitimer, Hertfordshire, UK).

### DC Block:

A constant-current cathodic DC blocking waveform was generated using a Keithley 2450 SourceMeter (Keithley, Cleveland, OH, USA).

### CSINE:

An electrode that allows for DC to be delivered safely to a nerve for extended periods of time has been described previously [1, 5]. Briefly, a high-capacitance carbon slurry was connected via a wire to the current source, and via a saline-filled tube to the nerve. A subcutaneous hypodermic needle in the lower back of the animal served as an electrical return for the CSINE. A custom designed nerve cuff was 3D printed in flexible resin (Flexible 50A resin, Form3B printer, Formlabs, Somerville, MA, USA).

### Data acquisition:

A custom LabVIEW (NI, Austin, TX, USA) application was used to control the foot stimulation and the blocking waveform. The raw EMG signal was filtered digitally to remove DC offset and breathing artifacts (DC remove, 0.1 s time constant), full wave rectified, then smoothed with a 0.0001 s time constant. To assess C-fiber-related activity, the area of the filtered signal in the time window of 150–600 ms post stimulation was calculated [13, 15].

#### 2.1. Verification of experimental setup

DC as applied via the CSINE electrode is used to block nociceptive action potentials on the sciatic nerve. The effect of block on adjacent neural structures was verified by isolating nearby efferent pathways and confirming that they were unaffected by block application. Any block of the adjacent efferent portion of the reflex loop would invalidate the experiment. A branch of the sciatic nerve at the level of the piriformis muscle innervates the biceps femoris, as well as the semitendinosus and semi-membraneous muscles. This branch has been described and illustrated, yet not named [33, 34]. The sciatic branch that innervates the biceps femoris was stimulated at 40 Hz using a custom-made bipolar hook electrode (platinum electrode with insulation on the underside) resulting in tetanic activation that was used to identify the pathway [33, 34]. In two animals sensory foot stimulation was alternated with sciatic branch motor stimulation, each at 0.2 Hz. It should be noted that this frequency produces force twitches for the purpose of testing block as opposed to the tetanic frequency used for pathway determination. The parameters for sensory stimulation are previously described. The sciatic branch motor stimulation was 20 *μ*s, 0.7 mA. The alternating stimulations were continued through a period of complete block of the CFR (1–2 min at −2 mA), and the signals were compared before, during and after the block period.

#### 2.2. Block trials

##### 2.2.1. Determination of instantaneous BT

Two types of experiments were performed on separate groups of rats to investigate the *induction* and *recovery* phenomena. In each rat, the surgical preparation and the position of the electrode on the nerve resulted in a varying amount of block in each animal. In order to normalize the effect of these experimental variations, each experiment began by determining the instantaneous BT_0_, the minimum DC current required to instantaneously block all nerve conduction.

**Figure 2.**
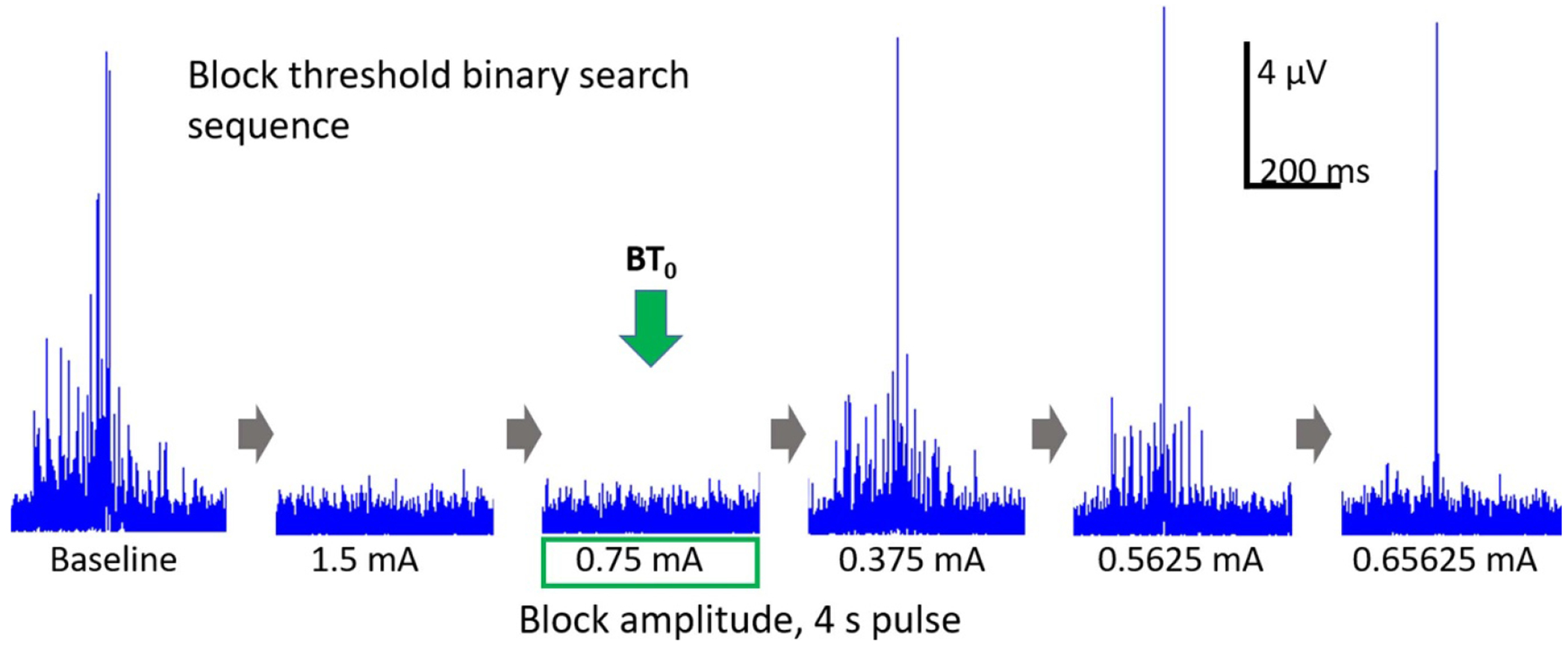
Example trials showing the determination of BT_0_: In this example the modified binary search algorithm was used to determine that 0.75 mA was the lowest DC block amplitude at which complete C-fiber conduction block could be achieved. This value is defined as BT_0_. Each trace shows EMG trace within the 150–600 ms window for C-fiber activation.

A 4 s pulse of cathodic DC block was delivered, starting 2 s prior to the foot stimulation. The amplitude of the DC was adjusted using a modified binary search to determine the minimum DC amplitude that would completely eliminate the EMG signal produced by the foot stimulation. The binary search algorithm assessed each block amplitude as either complete or incomplete block. Based on preliminary experiments the BT would be a maximum of −3 mA, therefore an initial value of −1.5 mA was examined. The search continued until a resolution of 20% of the tested value or 0.5 mA, whichever was smaller. An example BT_0_ trial is shown in figure 2.

##### 2.2.2. Procedures common to induction and recovery trials

Values for subsequent randomized trials were based on the BT_0_. Three statistical sets of amplitude or duration were used; each set was randomized. BT_0_ was reassessed before each set of trials. There was a wait time of 20 min between the end of the block period of each trial and the start of the block period of the subsequent trial [4].

It was noted that the determination of BT was affected by changes in anesthetic delivery. For this reason, a wait time of 20 min was also imposed between any change in anesthesia and subsequent trials. If it became necessary to adjust anesthetic levels during a statistical set of trials, this could result in a shift of the BT which was used to determine the test amplitudes for the set. In this situation, the set would be terminated, and any data would be omitted from analysis.

**Figure 3.**
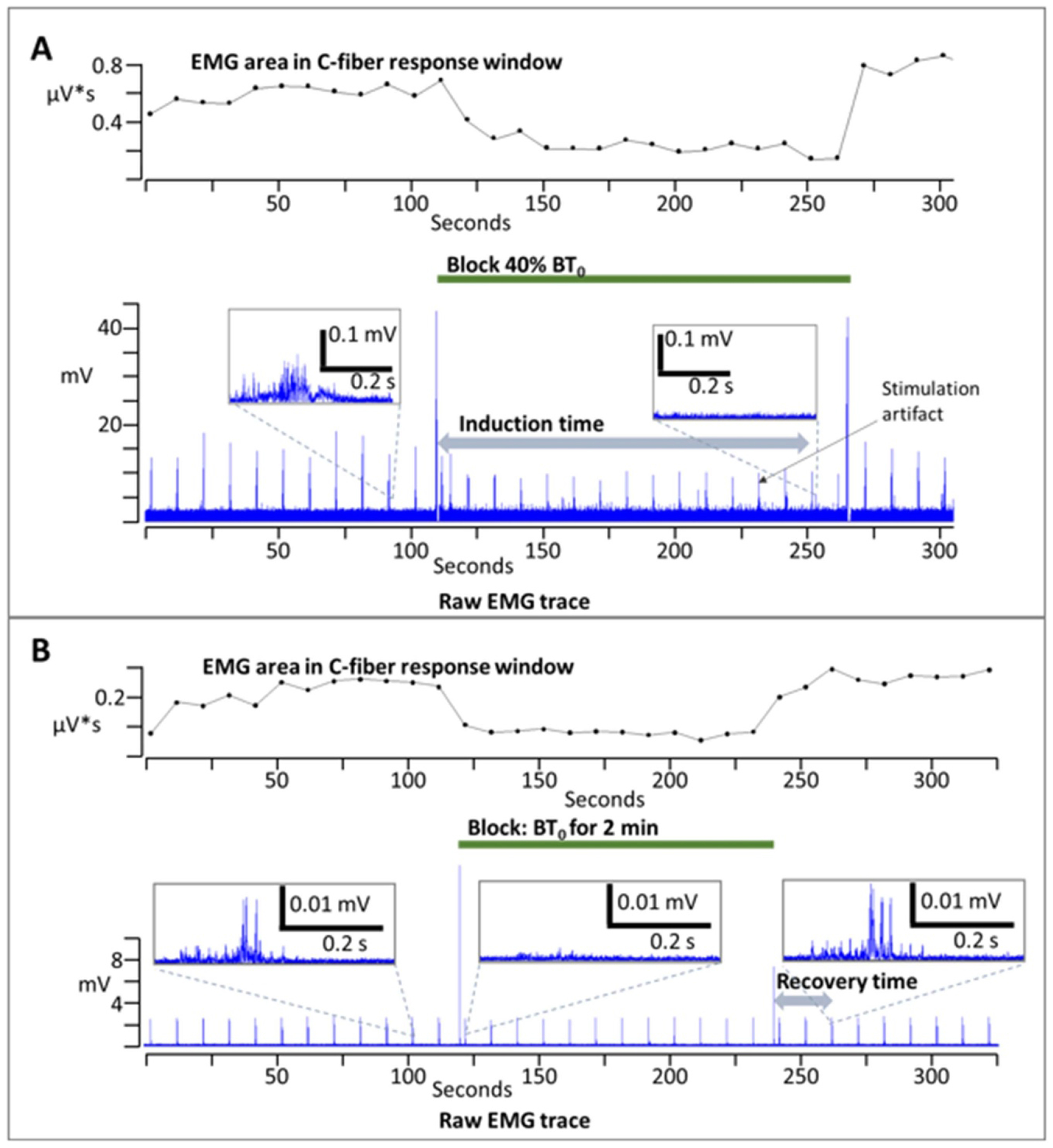
Example trials for induction time (A), and recovery time (B): recorded EMG activity over the entire trial is shown in the bottom trace for both A and B. Inset figures show the EMG trace during the C-fiber response window at various points throughout the trials. The top trace in A and B shows the integrated EMG area over the C-fiber range from 150–600 ms post stimulation at each trigger point.

Each trial (including trials to determine BT_0_) started with foot stimulation alone for 2 min to allow the EMG signal to reach plateau [35, 36], as seen in figures 3 (A) and (B). Temporal summation of C-fiber signals (also known as windup) occurs when a suitably high rate (>0.3 Hz) of C-fiber activation causes potentiation of the reflex-mediated motor output in the dorsal horn of the spinal cord [36]. In order to avoid the windup phenomenon, the frequency of foot stimulation was 0.1 Hz. Foot stimulation then continued through the block period and continued until the EMG signal recovered to baseline area in the 150–650 ms C-fiber window.

##### 2.2.3. Induction experiment

Block amplitudes of either 40, 60, or 80% of the instantaneous BT were applied continuously until complete block was achieved, at which point the block was turned off. The time until complete block (‘induction time’) was recorded in seconds. A limit of 300 s to achieve complete block was allowed for each trial.

##### 2.2.4. Recovery experiment

The instantaneous BT level was applied for a randomized time of 1, 2 or 3 min. Following the cessation of block, the recovery time was determined to be the first EMG signal that was equal to or greater than baseline area. Baseline area was the mean value of the 3 EMG signals prior to block onset.

##### 2.2.5. Statistical analysis

Both linear mixed models and linear regression models for induction time and recovery time were used, incorporating appropriate parameters of interest as fixed effects. ‘Animal’ and ‘set’ were included as random effects to rule out the effect of the preparation or trial dependent effects on the outcome. All statistical analyzes were carried out using R Statistical Software (v 4.3.2; R Core Team 2021).

## 3. Results

### 3.1. Verification of block localization

Due to the proximity of efferent pathways adjacent to the block electrode, it is possible that current leakage from the block electrode could affect the reflex motor pathway, masking the effect of block on the sensory pathway. It is important that any modulation of the CFR EMG signal be reflective of the block electrode’s effect on nerve transmission of the sensory action potentials in the sciatic nerve. Activation of the efferent response was achieved using bipolar hook electrodes. Stimulation at 40 Hz elicited tetanic contractions in the biceps femoris, giving visual and tactile confirmation of biceps femoris activation.

**Figure 4.**
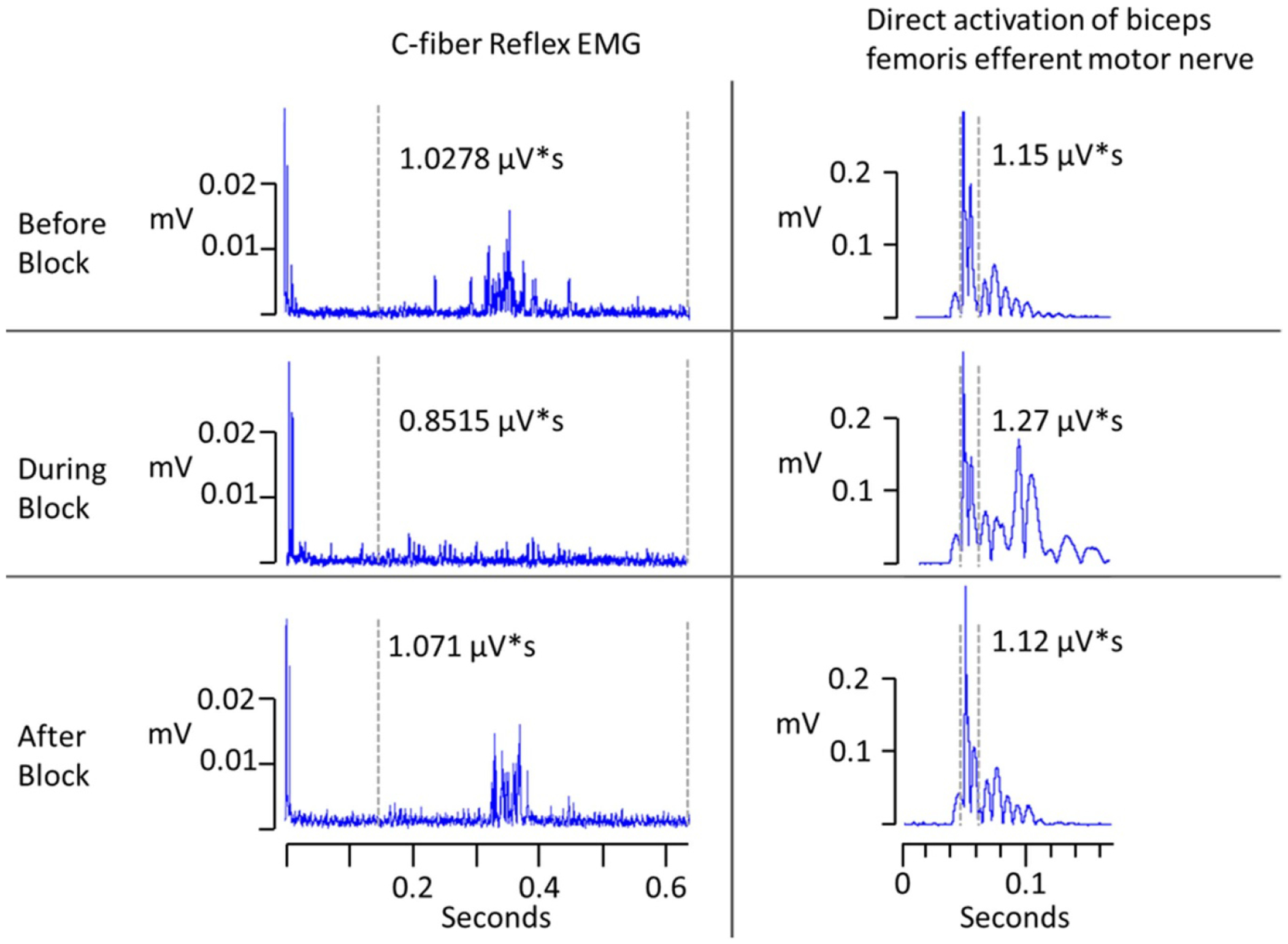
Electrophysiological validation: EMG activity is shown before, during and after block. Traces on the left demonstrate the response for afferent stimulation, while traces on the right show the response for efferent stimulation. The direct activation of the biceps femoris (right) is unaffected by the blocking waveform while the afferent response (left) is completely attenuated during block. This shows that the DC amplitude of −2 mA completely blocked the C-fiber reflex EMG, while the direct motor nerve EMG was unaffected.

In two animals, both the afferent and efferent sciatic pathways were stimulated alternately to determine the potential for confounding effects in the efferent pathway due to the block electrode. Stimulation alternated between the foot (eliciting the CFR EMG) and the hook electrode (eliciting an EMG response reflective of direct motor input to the biceps femoris). The efferent activation of the biceps femoris produced two peaks between 1.5–6.5 ms. This is consistent with the conduction velocity of motor fibers traveling approximately 40 mm [37, 38] with the additional delays as the signal passes the neuromuscular junctions and propagates through the muscle [39, 40]. This is in contrast to the C-fiber response that occurs from 150 to 650 ms. The example in figure 4 shows that the CFR EMG signal is completely blocked by application of −2 mA DC. In contrast, the EMG peaks resulting from direct motor nerve activation are not diminished by the application of DC block. This demonstrates that the efferent portion of the reflex loop is unaffected by the blocking current.

### 3.2. EMG Reflex sensory blocking results

Complete block of C-fiber action potentials in the sciatic nerve was achieved in all 13 experimental animals. The range of BT_0_ across all induction and recovery trials was 0.4687 to 3.75 mA. Given this variability, it was essential to analyze the effect of both absolute applied block levels as well as the percentage of BT_0_ in order to accurately elucidate the temporal effects of DC block. The potential effect of BT_0_ as a significant factor was investigated for each experiment described below.

### 3.3. Induction time results

**Figure 5.**
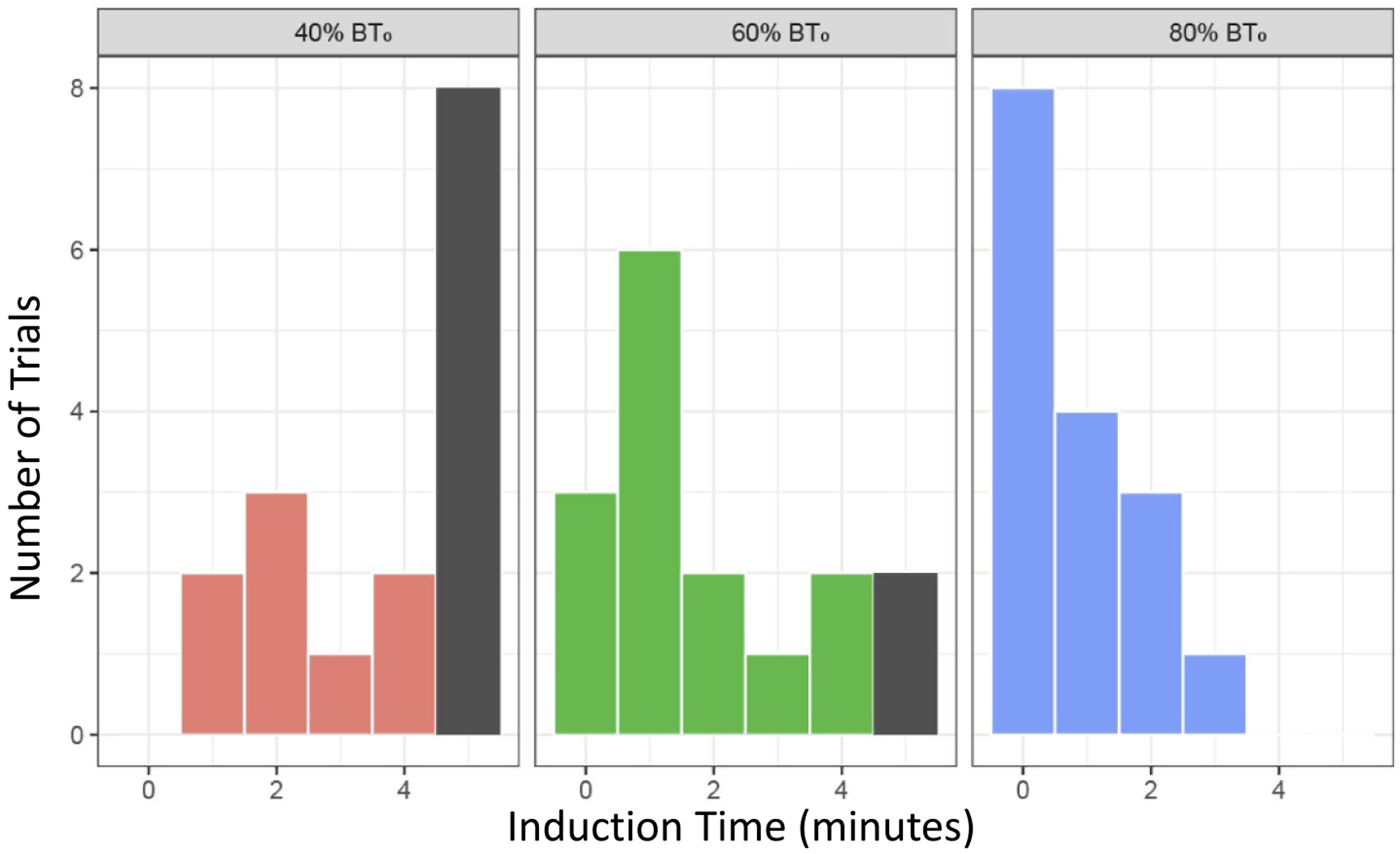
Histogram of complete block for each induction time: The number of trials that produced complete block within the 300 s time period are shown for each induction period. Trials are grouped by the percent of BT_0_ applied (*N* = 48) and binned by the induction time. Ten trials (dark grey bars) did not achieve complete block within the 300 s time limit. As the percent of block threshold is increased, the number of incomplete blocks and the induction time is reduced.

Data from 48 trials were collected from 7 animals to assess induction time. For every trial that does not block instantly, a period of partial block occurs before complete block is achieved [1]. However, we constrained our total trial time to 300 s in order to ensure complete data sets could be collected in a timely manner; 10 trials did not achieve complete block within the 300 s time limit. Figure 5 shows histograms grouped by the percent of BT_0_ applied. As the %BT_0_ increases, the number of incomplete blocks and the induction time are reduced, with no incomplete blocks at 80% BT_0_.

**Figure 6.**
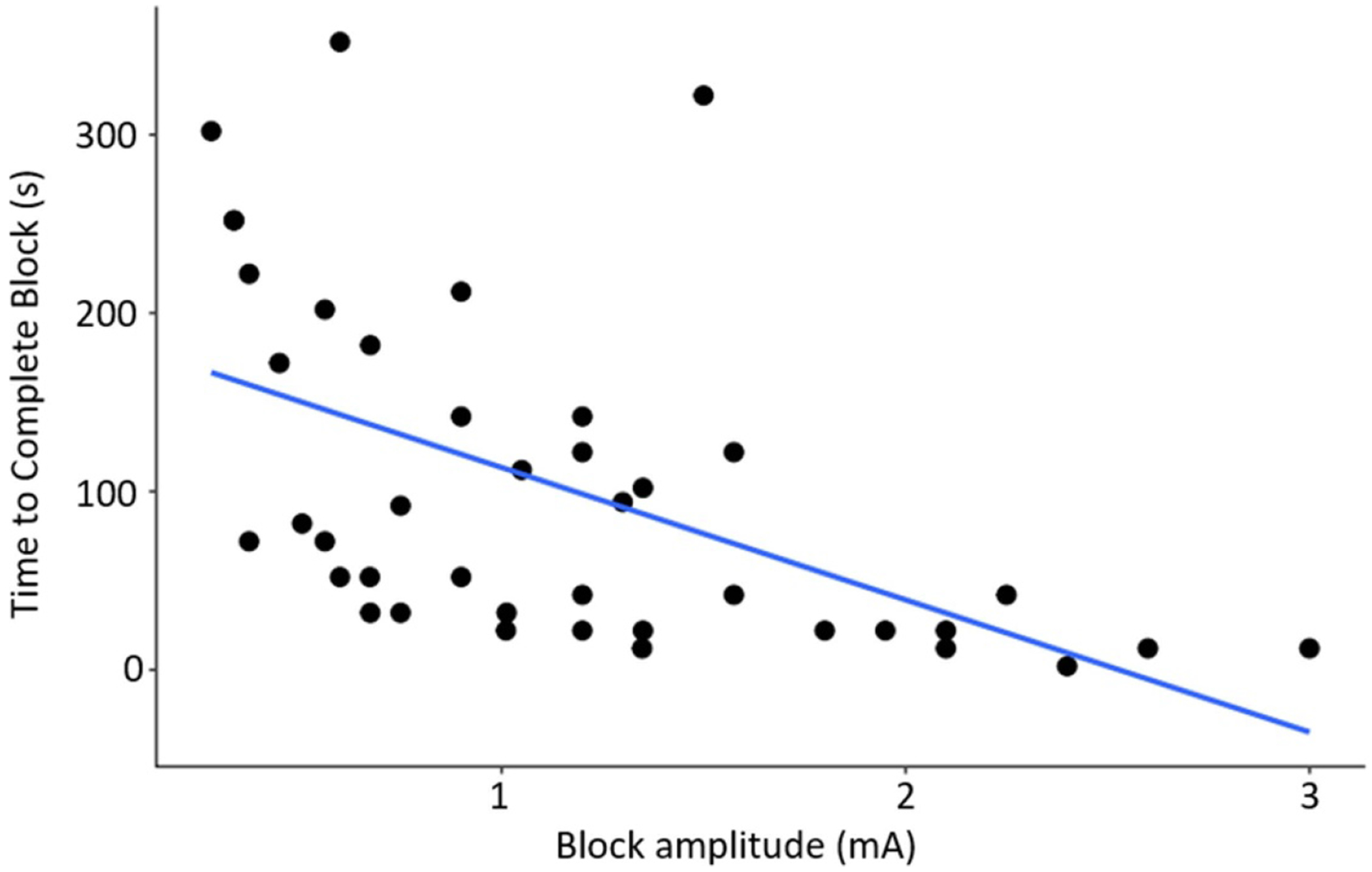
Effect of block amplitude on induction time: The amount of time required to achieve complete block (induction time) is compared to the amplitude (mA) of the applied block. Decreased current amplitude is correlated with longer induction times. Induction = − 64.6 × block amplitude + 160.8, *p* = 7 × 10–5, *R*^2^ = 0.36, *N* = 38 trials that achieved complete block.

The block amplitude in mA is determined by the product of the BT_0_ × %BT. This variable was plotted vs the induction time in figure 6 (excluding non-blocking trials). Among the 38 trials where a complete block was achieved, a lower block amplitude was associated with larger times to reach complete block. Specifically, an increase of 1 mA in absolute block value was associated with a decrease of 64.6 s (95% CI: 35.4, 93.8). The equation of the fit line is Induction time = − 64.6* block amplitude + 160.8, *p* = 7 × 10^−5^, *R*^2^ = 0.36. Although the figure appears to have a bimodal distribution, this is not an experimental bias but rather a result of the animal specific BT_0_ needed to achieve full block in each animal.

The %BT was used during the experiments to normalize for BT_0_ variations. This produced a significant effect (*p* = 0.012). However, using only BT_0_ also gave a significant effect (0.004). When both BT_0_ and %BT_0_ are included in the model, the *p* value is < 0.0001, indicating that each of the independent variables contribute meaningfully to the induction effect. The general linear mixed models for time to complete recovery included animal and set as random effects. Animal and set do not significantly contribute to any model.

### 3.4. Recovery time results

**Figure 7.**
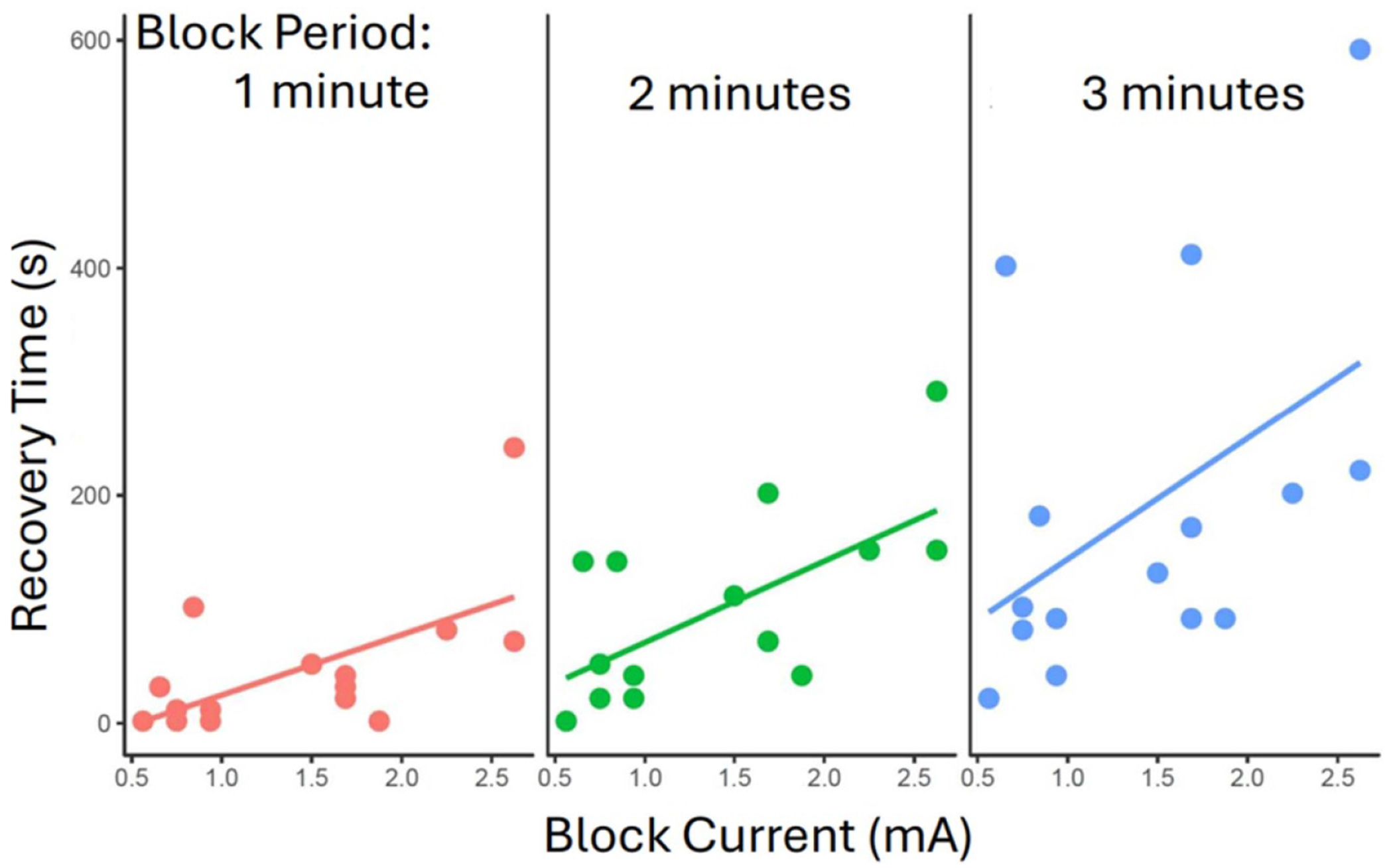
Comparison of block current to recovery time for different periods of block: block was applied at different currents for durations of 1, 2 and 3 min. The amount of time required for the EMG activity to be completely restored was recorded (recovery time) for each block period, the recovery time increases as the block current is increased. *N* = 45 trials.

Data from 45 trials were collected from 6 animals to assess recovery time. BT_0_ current, producing 100% block in each animal, was delivered for set time periods of 1, 2, and 3 min (figure 7). An increase in the block period resulted in an increase in the recovery time (linear regression coefficient = *X; p* = 0.004; *R*^2^ = 0.235). Likewise, BT_0_ is positively correlated with time to recovery (linear regression coefficient = 76.91; *p* = 0.003; *R*^2^ = 0.190). Simple linear models that include both BT_0_ and block period to predict time to recovery perform better than univariate models (*R*^2^ = 0.425), and ANOVA analysis between a model which only uses BT_0_ compared to a model that includes both BT_0_ and block period indicates that including block period adds meaningful predictive value (*p* < 0.001).

**Figure 8.**
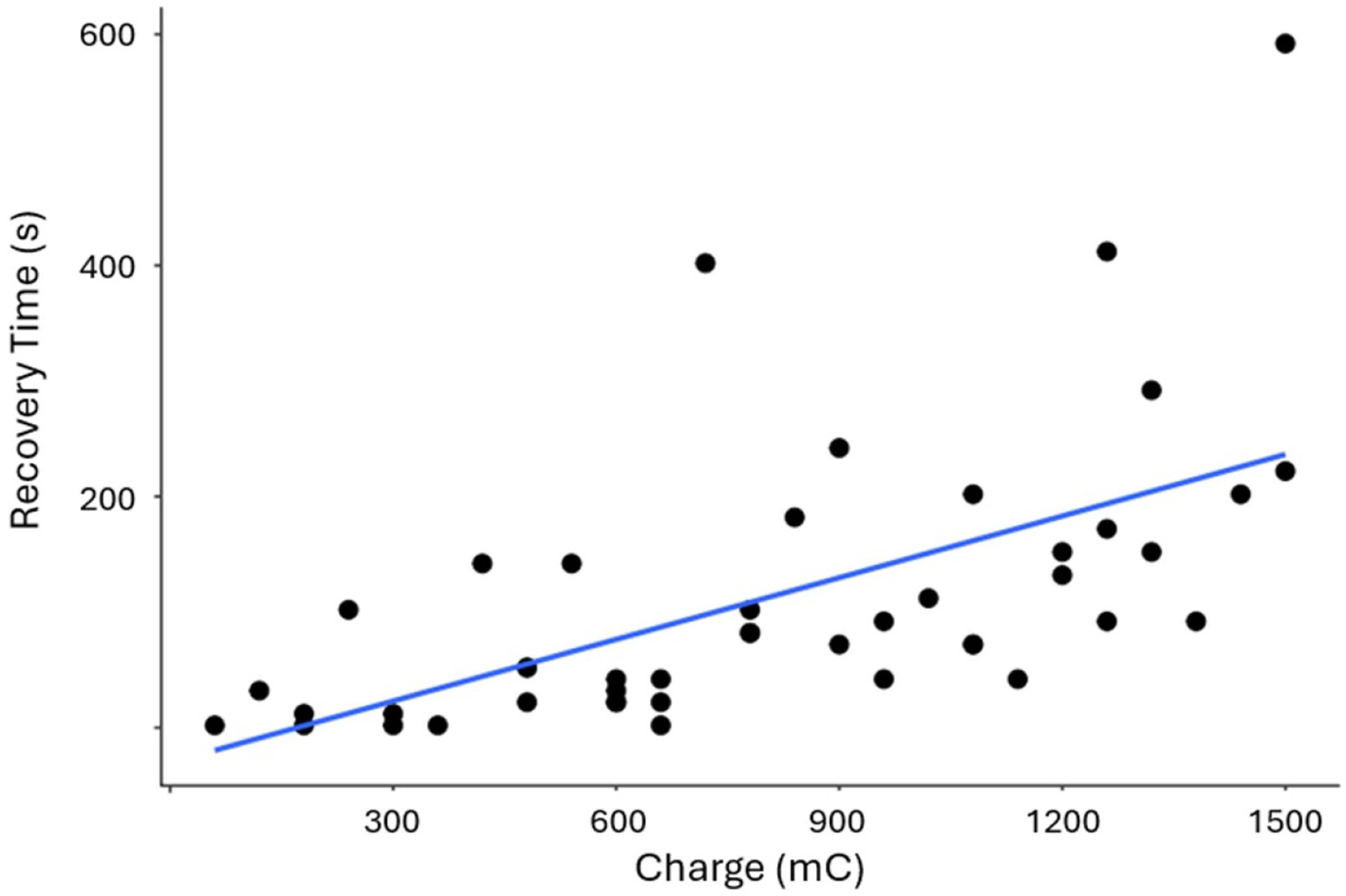
Effect of charge on recovery time: The amount of charge (mC) for each trial is defined as the amount of current multiplied by the duration of the trial in seconds. This charge injection is compared to the recovery time. Increased charge is correlated with increased recovery time. Recovery = 0.7 × charge −6.4, *p* = 1.3 × 10^−6^, *R*^2^ = 0.42, *N* = 45 trials.

The charge in mC is determined by the product of the BT_0_ x block period. This variable was plotted vs the recovery time in figure 8. A higher charge was associated with longer times to reach recovery from block. Specifically, an increase of 1 mC in charge was associated with an increase of 41.8 s (95% CI: 26.8, 56.8). The equation of the fit line is recovery = 0.7 × charge −6.4, *p* = 1.3 × 10^−6^, *R*^2^ = 0.42.

The general linear mixed models for time to complete recovery included Animal and Set as random effects. Animal and Set do not significantly contribute to any model, and models including both BT_0_ and block period (either independently or combined as charge) fit the observed data better than those which include only one of these measures (table 1).

## 4. Discussion

In this study, the temporal properties of DC nerve block were tested in experimental pain model. The goal of this work was to determine the viability of block for pain of peripheral origin, as well as characterize the dosage needed to provide effective pain relief. In addition, the temporal responses provide insight into possible mechanisms of DC nerve block that can be further explored in modeling or additional *in-vivo* experiments. Using computer modeling of a myelinated axon, it has been suggested that the temporal effects of DC block are caused by accumulation of extra axonal potassium in the extracellular space [7]. This study provides evidence that the temporal effects of block are significantly affected by charge delivered as opposed to cessation of activation of the nerve. This points to an ionic mechanism as opposed to a metabolic mechanism for both the induction and recovery effects. Further research would be useful to elucidate this matter.

Complete block of sensory C-fiber activity was achieved in all 13 animals. Without exception, the nerve conduction block was completely reversible, as evidenced by a return to baseline EMG area amplitude. In the induction experiments a majority of the lowest %BT_0_ (40%) value were able to achieve complete block within the testing period. This is promising for clinical applications as lower currents would be generally perceived as safer and more power efficient. This is especially useful in the sensory system because, in contrast to the autonomic system [5], fine temporal precision is not critical. The induction phenomenon, described in the introduction, has been characterized in the rodent motor and autonomic systems [1, 4, 5], and demonstrated in the rodent sensory system [7]. The data in the present study show that the relationship of induction time to block amplitude of C-fibers is comparable to that shown in the motor and autonomic systems: induction time increased as %BT decreased.

In the recovery trials, the EMG signal always returned to baseline levels, suggesting that the delayed recovery is not caused by any permanent damage to the sciatic nerve. Examples of the recovery effect have been shown in the rodent sensory system for all classes of sensory fiber [4, 6, 7]. To date, this the sensory recovery effect has not been systematically studied. However, it has been studied systematically in the rodent motor and autonomic systems [1, 5]. In agreement with the results from the present study, in the motor and autonomic systems it was shown that higher charge in a trial led to longer recovery times, be it from higher currents for a shorter time or lower currents for a longer time [1, 5]. The recovery phenomenon is also of potential benefit in a clinical application since a device could be switched off intermittently to increase safety margins and, in the case of an implantable device, save power output [41–45].

Although a linear model of the recovery is presented here, exponential and polynomial models were also assessed, with the linear model providing the optimal fit. Several possible mechanisms could contribute to the recovery effect: sodium-potassium pump dynamics or K+ accumulation could result in a different fit such as an exponential model, but this is not demonstrated by our data. However, this does not rule out the possibility of these factors for the mechanisms of induction or recovery, and the combination of these factors may result in the linear relationship shown here. There have been several modeling studies looking at the mechanisms of the carry over effect for kilohertz frequency alternating current (KHFAC) block [41–45]. Although the basic mechanisms of DC block have been modeled [2],there hasn’t been a published modeling study specifically for the recovery effect. Modeling of combined DC and KHFAC waveforms have shown how the recovery effect can be leveraged to inhibit the onset activity of KHFAC [46],but this model examined a shorter, transient recovery effect as opposed to the persistent effect seen in these *in vivo* experiments. Initial modeling efforts in our lab point to potassium accumulation as a possible mechanism for recovery and will be the subject of a future publication.

BT_0_ was determined before every randomized set of trials in order to quantify and normalize for the variability in animals, surgical procedures, and electrode placements. By using %BT (induction experiments) or BT_0_ for set time periods (recovery experiments) we were able to set current and charge values within an appropriate dynamic range to observe the temporal properties of the electrode-nerve interactions. It is interesting, therefore, that BT_0_ (induction) and charge (BT_0_ × time; recovery) were also statistically significant, adding to the predictive value. The precise interactions between these factors warrants further study.

The EMG responses associated with CFR have been used extensively to study pain responses in animals and humans, as well as to study the effects of systemically administered agonists and antagonists of pain [13, 16, 18, 20, 47]. To date, this method has not been used to test the efficacy of an electronic device, and it was therefore necessary to validate the technique for such use. The success of this validation opens the possibility for its use in device testing in future research.

Clinically, it would be advantageous, especially in chronic pain conditions, if it were possible to block unmyelinated nociceptive C-fibers at lower DC currents than for myelinated A-fibers. Such a situation would enable the preservation of tactile sensory feedback to allow normal movement whilst eliminating pain. Previous *in-vivo* studies disagree as to whether this order of selective block is possible [6, 7]. If a strategy is possible then complete C-fiber block would be preferable. If not, then perhaps a partial block of C-fibers could strike a balance between reducing pain whilst preserving tactile sensation and voluntary movement.

Under physiological conditions C-fibers do not show any spontaneous activity [48]; however, in neuropathic pain conditions overactive C-fiber firing can lead to central sensitization. These neuroplastic changes in the spinal cord lead to an amplification of the nociceptive inputs, and bilateral hypersensitivity pain syndromes [30, 49, 50]. This pathological C-fiber activity is requisite for perpetuating central sensitization, and in rodent studies, blocking this activity unilaterally with a pharmacological agent can reduce bilateral hypersensitivity. In a clinical application, even if blocking C-fiber activity causes concurrent block of tactile sensitivity, it may be worthwhile as an intermittent therapy in order to prevent pain the onset of hypersensitivity or lessen the severity of existing conditions.

## 5. Conclusion

The temporal properties of DC nerve block of nociceptive C-fibers in the rat were investigated. An established *in-vivo* model for the investigation of pain and pain pharmacotherapies was used in a novel way to allow assessment of an electrical device. The induction and recovery phenomena show similarities with the motor and autonomic system. At amplitudes below the BT, complete block could be induced over a period of seconds to minutes, with lower currents being correlated with longer induction times. When block was applied for prolonged periods of time, block was sustained following the cessation of DC delivery. Recovery was longer for longer durations of block application. These phenomena could be leveraged to improve safety and efficacy of DC nerve block in future clinical applications to treat both acute and chronic pain syndromes. Further investigations, possibly using computational models [51] and ex-vivo nerve preparations [52],would be an important step towards understanding of the cellular and molecular mechanisms of the temporal properties of DC nerve block.

## Figures and Tables

**Table 1.** Summary statistics of general linear mixed effects models for time to recovery (minutes).

Fixed effect parameters	Animal Wald *p*-value	Set Wald *p*-value	BT_0_ (mA) *p*-value	Block period (min) *p*-value	Charge (mC) *p*-value	F Ratio
Block period	0.368	0.511	—	<0.001	—	19.41
BT_0_	0.363	0.534	0.005	—	—	9.40
Block period, BT_0_	0.237	0.512	<0.001	<0.001	—	28.76 (Block period), 16.46 (BT_0_)
Charge	0.228	0.509	—	—	<0.001	47.16

## Data Availability

The data that support the findings of this study will be openly available following an embargo at the following URL/DOI: https://dataverse.harvard.edu/dataset.xhtml?persistentId=doi:10.7910/DVN/ODBHZY [53]
